# A New Treatment for Burns and Frost-Bites

**Published:** 1888-03

**Authors:** 


### *A NEW TREATMENT FOR BURNS AND FROST-BITES.*

In *Monde de la Science et de l'Industrie,* Buboff treated successfully sixty cases of burns and frost-bites by his method, which consists in the application of compresses of cotton or linen moistened in a solution of permanganate of potassium. The strength of the solution is from one to three grains to an ounce of water. The remedy is only efficacious in frost-bites of the first or the second degree, and in burns of the first degree.

In every case, the pain rapidly subsided, and the inflammation was relieved; when the blisters were unruptured, suppuration was always prevented. The doctor cites the case of a patient who was burned over nearly the whole surface of the trunk while taking a vapor bath; the permanganate solution relieved the pain in an hour; the epidermis exfoliated, and recovery was complete at the end of a week.

